# Room temperature, cascadable, all-optical polariton universal gates

**DOI:** 10.1038/s41467-024-49690-3

**Published:** 2024-06-25

**Authors:** Denis A. Sannikov, Anton V. Baranikov, Anton D. Putintsev, Mikhail Misko, Anton V. Zasedatelev, Ullrich Scherf, Pavlos G. Lagoudakis

**Affiliations:** 1 Hybrid Photonics Laboratory, Skolkovo Institute of Science and Technology, Territory of Innovation Center Skolkovo, Bolshoy Boulevard 30, building 1, 121205 Moscow, Russia; 2 Macromolecular Chemistry Group and Institute for Polymer Technology, Bergische Universität Wuppertal, Gauss-Strasse 20, 42119 Wuppertal, Germany

**Keywords:** Bose-Einstein condensates, Polaritons, Nonlinear optics

## Abstract

Today, almost all information processing is performed using electronic logic circuits operating at several gigahertz frequency. All-optical logic holds the promise to allow for up to three orders of magnitude higher speed. Whereas essential all-optical transistor functionalities were demonstrated across a range of platforms, utilising them to implement a complete Boolean logic gate set and in particular negation, i.e. switching off an optical signal with another, weaker, optical signal, poses a major challenge. Here, we realize a cascadable NOT gate by introducing the concept of non-ground-state polariton amplification in organic semiconductor microcavities under non-resonant optical excitation. We unravel the importance of vibron-mediated stimulated scattering in room temperature operation of the inverter. Moreover, we extend the concept to a multi-input universal NOR logic gate, where in the presence of any of the input signals non-ground-state amplification supersedes spontaneous ground-state condensation, resulting in a NOR gate with ~1 ps switching time. The realisation of an ultrafast universal logic gate constitutes an essential step for more complex optical circuitry that could boost information processing applications.

## Abstract (web summary)

A cascadable all-optical NOT gate is a requirement for full-logic in optical computing. By introducing the concept of non-ground-state polariton amplification in organic semiconductor microcavities, the authors realized the operation of an all-optical cascadable universal gate.

## Introduction

While the number of transistors that make up a processor has been growing exponentially over the last five decades the clock speed stalled at a few gigahertz about 15 years ago^1^ as a result of the breakdown of Dennard scaling^2^. The key challenge is to keep the power density constant while further reducing dynamic dissipation when the clock frequency is increased. Current state-of-the-art transistors, although scaled down to single nanometer dimensions, typically require several attojoule switching energy. More energy-efficient electronic devices, like single electron transistors have been investigated but were found to be incompatible with high speed, room temperature operation and established processing methods. Earlier attempts on single photon all-optical transistors, based on epitaxially-grown quantum dots in photonic crystals, offered sub-attojoule switching energies^3,4^ but were limited to cryogenic operating temperatures, and faced similar roadblocks as their electronic counterparts, in addition to unsolved paths for cascadability and scale-up. In principle, optical circuits could offer reduced energy consumption, whilst increasing switching speeds, and also harness a more precise clocking compared to electronics^5,6^. However, density scaling of optical circuits is limited, and probably will hardly ever reach the device densities of modern integrated electronics. Instead, simpler circuits compared to full-fledged microprocessors, could run at orders of magnitude higher speed, while maintaining low power dissipation, and thus revolutionize data processing tasks that are today relying on application-specific integrated circuits (ASICs) or field-programmable gate arrays (FPGAs)^5^.

A common architecture of all-optical transistors involves an optical resonator—cavity, photonic crystal or other—and a nonlinear optical process in the form of a higher order susceptibility, or an optical resonance in atomic gases or semiconductors^7–10^. As such, semiconductor microcavities offer an apparent all-optical transistor platform using excitons strongly coupled to a cavity mode resulting in the formation of hybrid light-matter eigenstates called exciton-polaritons^11,12^; hereafter polaritons. Polariton transistor operation, including switching^8,13,14^, amplification^15,16^, and some logic gate functionality^8,17^ were demonstrated in III-V semiconductor microcavities, alas at cryogenic temperatures. Recently, room temperature all-optical transistor operation was demonstrated in strongly coupled organic semiconductor polymers embedded in optical microcavities with sub-picosecond switching time, a record net gain of ~10 dB *μ*m^−1^, cascadable all-optical AND/OR logic gate functionality^10^, as well as single photon switching^18^. However, a cascadable NOT gate, the essential ingredient for complete logic, has still remained elusive^19^. Polariton transistors have exploited the signal amplification that occurs through stimulated scattering to the ground polariton state, in the presence of a resonant seed beam^8,10,15,16^, leading to a macroscopic coherent wavefunction. The next logical step for the realization of the NOT gate, i.e. switching off ground-state condensation with an external optical beam has been previously realized with coherent control over the polariton wavefunction^20^. Here, we circumvent the necessity for coherent control of ground-state polariton condensation by introducing the concept of non-ground-state polariton amplification that switches off the ground-state emission. Although, optical switches controlling polariton flows in waveguides have been realized through the injection of a potential barrier impeding the propagation of polaritons^8,13,21^, the absence of cascadability has prevented their implementation in full logic circuitry.

In this work, we theoretically explore the conditions under which non-ground-state amplification occurs by means of a microscopic model^22^. We apply our theoretical prediction and experimentally realize non-ground-state amplification using a control beam, resonant to a higher energy and in-plane momentum polariton state that results in the depletion of the ground-state polariton condensate. The above configuration constitutes the operation of a NOT gate, whose output is the emission from the ground-state polariton condensate. We exploit the two-dimensional parabolic shape of the lower polariton dispersion and extend the NOT gate to demonstrate a polariton universal logic gate operating at room temperature, with ~1 ps switching time and up to 8 parallel inputs.

## Results

Under non-resonant optical pumping, polariton condensation is driven by the rapid thermalization of carriers to the polariton ground state^22^. When thermalization exceeds polariton losses, it can result in occupation numbers that trigger spontaneous symmetry-breaking and the emergence of a macroscopic polariton wavefunction^12^. In the presence of a resonant scattering process, simultaneous seeding of the polariton ground state with a resonant probe beam, may result in polariton amplification. Polariton amplification was observed in inorganic microcavities through resonant pair-polariton scattering^15,16^, and more recently in organic microcavities^10^ via resonant exciton-vibron scattering^23^. In the absence of any resonant scattering process, polaritons injected resonantly at a non-ground-state of the lower polariton dispersion thermalize towards the ground-state rendering non-ground-state amplification, unattainable^22^. From a microscopic perspective, for non-ground-state amplification to occur, a resonant scattering process should exist that has a higher transition rate than thermalization towards the polariton ground-state. In non-crystalline organic semiconductors, in the absence of exciton-exciton scattering, energy relaxation from the exciton reservoir to the lower polariton dispersion, and subsequently across the dispersion, occurs either through intracavity radiative pumping from uncoupled molecules, or through vibrons. We note that in non-crystalline organic semiconductor microcavities, exciton localization precludes exciton exchange interactions, the dominant mechanism for energy relaxation in inorganic crystalline semiconductor microcavities. In both inorganic and organic microcavities, exciton scattering with high energy LO-phonons, or vibrons resonant to the ground state was shown to be an efficient mechanism that results in faster energy relaxation and lowering of the threshold for polariton condensation^10,23,24^.

**Fig. 1:**
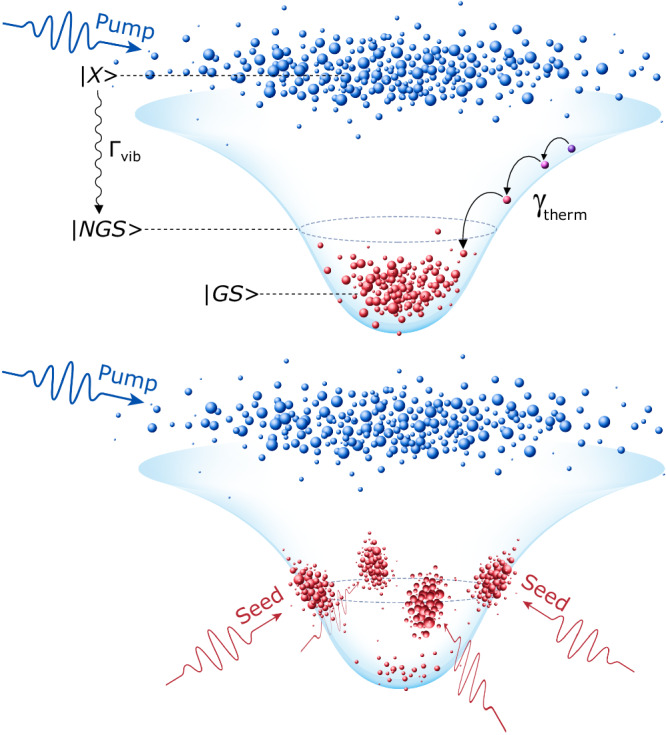
Non-ground-state polariton amplification. The schematic represents the paraboloid of the linear lower polariton dispersion branch. The pumping pulse (blue arrows) non-resonantly injects a hot exciton reservoir, $\left\vert X\right\rangle$, while thermalization processes populate the polariton ground state, $\left\vert GS\right\rangle$, that leads to polariton condensation through bosonic stimulation (red cloud of particles on the top schematic). In the presence of resonant seed pulses injecting polaritons at opposite in-plane wavevectors (*k*_∥_ ≈ ± 2.55 *μ*m^−1^) (red arrows), the rate of vibron-assisted transitions, Γ_vib_, into the seeded modes dominates the thermalization rate, *γ*_therm_, stimulating non-ground state amplification, $\left\vert NGS\right\rangle$, and depleting the ground-state.

**Fig. 2:**
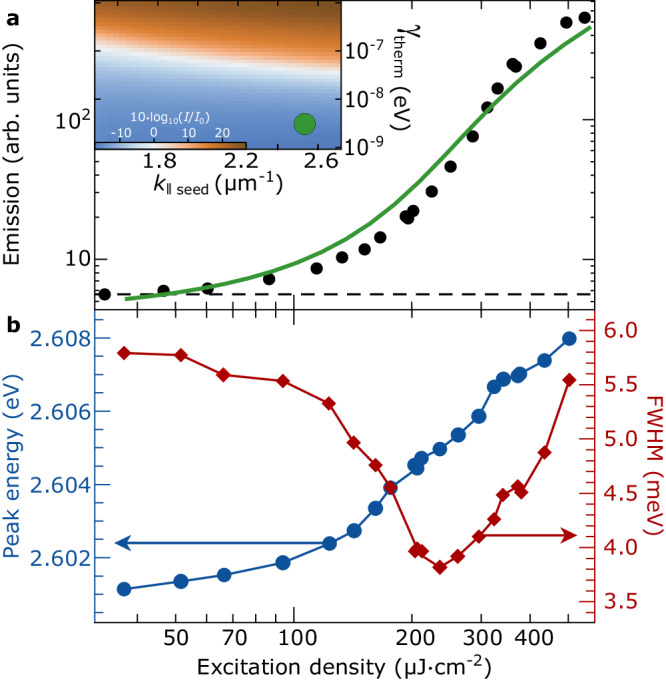
Spectroscopic characterization of non-ground-state polariton amplification. **a** Emission from *k*_∥_ ≈ − 2.55 *μ*m^−1^ angularly integrated over ~*k*_∥_ ∈ (−2.96;−1.86) *μ*m^−1^ versus pump excitation fluence at zero pump-seed time delay. The horizontal dashed line represents the level of the recorded intensity from the seed-only. The solid green curve shows the numerical results of non-ground-state polariton amplification. **b** Emission photon energy at the maximum of the emission spectrum (left/blue axis) and full-width at half-maximum (FWHM, right/red axis) versus pump excitation fluence at zero pump-seed time delay. The color-map inset on panel (**a**) shows the numerically calculated ground-state condensate depletion in the presence of the seed for different intra-band thermalization rates (vertical axis) and in-plane momenta of the seeded mode (horizontal axis). The blue area corresponds to the parameters that allow for ground-state condensation depletion. The green dot represents the best fit to the experimental observations.

In the limit of fast thermalization and considering both the optical pump, the optical seed and energy relaxation in the Born–Markov approximation, we model the polariton dynamics across the lower polariton dispersion by solving the master equation in the Lindblad form for the full polariton density matrix, $\hat{\rho }$^22^. In the presence of a high-energy vibron mode, tuned in resonance with the optically seeded non-ground-state, we obtain

1 $$\frac{{{d}}\hat{\rho }}{{{d}}t}=\frac{i}{\hslash }\left[\hat{\rho },{\hat{H}}_{T}\right]+{\hat{L}}_{T}(\hat{\rho })$$

where ${\hat{H}}_{T}$ is the sum of the polariton Hamiltonian, neglecting pair polariton scattering, and the Fröhlich Hamiltonian for the vibron mode and a standard optomechanical Hamiltonian for the exciton-vibron interactions. ${\hat{L}}_{T}$ is the sum of the Lindblad superoperators for polaritons, vibrons, the pump, the resonant non-ground-state seed, and the thermalization of polaritons across the lower polariton dispersion; see Section I in the Supplementary Information (SI) for analytical expressions. Figure 1 depicts schematically the paraboloid of the lower polariton dispersion in the absence (top) and presence (bottom) of the seed pulse, superimposed with the dispersionless hot exciton reservoir (blue-bubbles cloud) injected by the pump-pulse (blue arrow), wherein with the vertical wavy black arrow we indicate the vibron-mediated energy relaxation rate from the hot exciton reservoir to the lower polariton dispersion, Γ_vib_. Smaller curved black arrows annotate the intra-branch thermalization, *γ*_therm_. The non-ground-state seed pulses, resonant to the lower polariton dispersion, are shown by red arrows. In the inset of Fig. 2a, we plot the quenching of the ground-state polariton density in the presence of the resonant seed vs the intra-branch thermalization rate, *γ*_therm_, and the in-plane momentum of the seeded mode, see Section II in the SI for a complete description of the microscopic theoretical model. The blue area corresponds to the range of values wherein ground-state condensation depletion may occur. We note that in the absence of a resonant exciton-vibron transition polariton condensation cannot be realized^10^.

Next, we investigate experimentally the formation of non-ground-state polariton amplification using a semiconductor polymer microcavity consisting of methyl-substituted ladder-type poly-[paraphenylene] (MeLPPP) sandwiched between distributed Bragg reflectors (DBR). Strong coupling of the cavity mode (2.64 eV) and two sub-levels of the first excited singlet state (S_10_ at 2.72 eV and S_11_ at 2.91 eV) result in three polariton branches (see Section I in the SI), with the paraboloid of the lower polariton dispersion shown in Fig. 1, exhibiting a Rabi splitting between the middle and the lower polariton branches of 2Ω_*R*_ = 170 meV^25^. Under non-resonant optical pumping at condensation threshold, the full-width at half-maximum (FWHM) of the ground-state polariton wavefunction in Fourier-space is ~0.49 *μ*m^−1^. In order to minimize the angular overlap between the ground-state polariton condensate (corresponding to the logic gate output signal) and the seed pulse (corresponding to the logic gate input signal), we tune the energy of the seed in resonance with the lower polariton branch at an in-plane wavevector of ~−2.55 *μ*m^−1^, as shown with red arrows in Fig. 1. We also tune the non-resonant optical pump at one molecular vibron above the energy of the seed pulse to enable a single-step vibron-mediated energy relaxation from the hot exciton reservoir, as shown with a long, vertical, black arrow in Fig. 1. For technical description of the experimental setup please see Section III in the SI. The non-resonant pump excitation density dependence of the emission intensity resonant to the seed and at zero pump-seed time-delay is shown in Fig. 2a. The horizontal dashed line indicates the transmission intensity of the seed-only pulse. The green solid line in Fig. 2a corresponds to the numerical results of the microscopic model, see Section I in the SI. Figure 2b displays the corresponding FWHM (in red) and the emission energy (in blue) at the maximum of the emission spectrum. At the threshold, we observe a decrease of the FWHM and an energy blue-shift, indicative of polariton amplification resonant to the seed^26^. We note that the linewidth narrowing in the case of polariton amplification is reduced by a factor of ≈2, in agreement with previous measurements of ground-state amplification^10^.

**Fig. 3:**
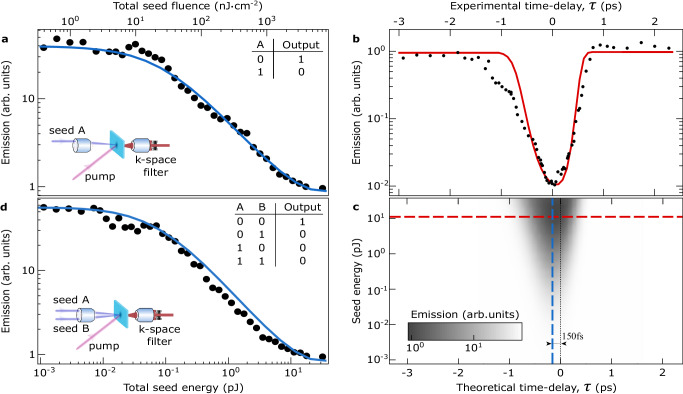
Depletion of the ground polariton state. **a**, **d** Ground-state condensate emission filtered over ~±1 *μ*m^−1^ versus seed energy and seed fluence at zero pump-seed time delay for one (**a**) and two (**d**) seed pulses. The seed pulses are of equal power and inject polaritons resonantly at opposite in-plane wavevectors (*k*_∥_ ≈ ± 2.55 *μ*m^−1^). The blue curves represent the best fit with the numerical model. The inset tables in (**a**) and (**d**) are the truth tables for the NOT and NOR gates respectively. **b** Ground-state condensate emission versus the time delay between pump and seed pulses (negative time delay is for seed-before-pump configuration). The top horizontal axis is centered so that the zero time delay corresponds to the maximum condensate depletion. The bottom horizontal axis represents the time delay as according to the numerical simulations. The red curve represents the results of calculations with the same best fit parameters. **c** The results of numerical simulations for various seed energies and pump-seed time delays. The gray-scale color corresponds to the filtered emission intensity from the ground state. The dashed lines correspond to the fitting curves from panels (**a**) (vertical dashed line) and (**b**) (horizontal dashed line) with the respective colors.

To evaluate the depletion of the emission intensity at the ground state, we examine the influence of the pulse energy of the seed beam. In the absence of the seed pulse and at an excitation density approximately 2*P*_th_, we observe all the hallmarks of ground-state polariton condensation, see Section IV in the SI. Figure 3a shows the emission intensity from the ground-state polariton condensate angularly filtered at normal incidence, *k*_∥_ = 0, with a width of ~ 2 *μ*m^−1^, versus seed beam energy for zero pump-seed time-delay. The pump excitation density is kept constant at *P* ~ 2*P*_th_ corresponding to the pump saturation regime in order to minimize the noise induced by laser intensity fluctuations. With increasing seed pulse energy the emission intensity decreases monotonically. This monotonic dependence is also predicted by the theoretical model, the numerical results of which are shown with a solid blue line in Fig. 3a. In the presence of the seed pulse and for the parameters corresponding to the green dot in the inset of Fig. 2a, we observe that polariton relaxation to the non-ground-state is more efficient than to the ground-state, resulting in −17 dB depletion of the ground-state emission. The quenching of the ground-state condensate in the presence of the seed corresponds to the NOT gate functionality, where the pump beam charges the transistor and the seed beam acts as the control input that switches the output between “1” and “0” states.

Further on, we investigate the dependence of the ground-state condensate quenching on the time delay between the arrival of pump and control-seed pulses, shown in Fig. 3b. In the experimental configuration we consider that the maximum switching contrast corresponds to the zero time-delay between the pulses. However in the numerical simulations (solid red curve) the maximal depletion of the condensate occurs when the control pulse arrives approximately 150 fs prior to the pumping pulse creating a more efficient preoccupation of the mode. The theoretical model allows us to vary simultaneously both the time-delay and the control-seed beam energy. The results of the ground-state condensate quenching are presented in Fig. 3c. The colored dashed lines depict the corresponding fitting curves presented in Fig. 3a, b respectively. An important advantage over previous realizations of polariton AND/OR gates^10^ is that the repetition rate of the NOT gate is not thwarted by the relatively slow dynamics when no condensate state is reached, i.e. for a logic “0” output. In the NOT gate, a macroscopic occupation of a polariton state is always present independently of the logic levels, formed at distinctly different wavevectors for “0” and “1” through the rapid depopulation of the exciton reservoir, i.e. with ~1 ps response time.

Building on the concept of non-ground-state polariton amplification, we demonstrate a universal NOR gate. We configure a two-input NOR gate by adding a second seed beam injecting polaritons resonantly with an opposite in-plane momentum, i.e. *k*_∥,*B*_ = − *k*_∥,*A*_, as shown schematically in Fig. 3d keeping the pulse energy of the two seed beams equal. Figure 3d shows the emission intensity from the ground-state polariton condensate, angularly filtered at normal incidence, *k*_∥_ = 0, with a FWHM of ~2 *μ*m^−1^ versus seed energy for zero pump-seed time-delay. Similarly, to the single seed beam experiment of the NOT gate, with increasing the seeds’ pulse energy we observed a threshold, depicted with a gray shaded area, above which the emission intensity also decreases monotonically. The solid blue line shows the numerical results of the microscopic theory in the presence of the two seed beams. This two seed beam configuration exhibits the depletion of the ground-state emission of −18 dB. Evidently, the two seed beams act as control inputs to the NOR gate.

**Fig. 4:**
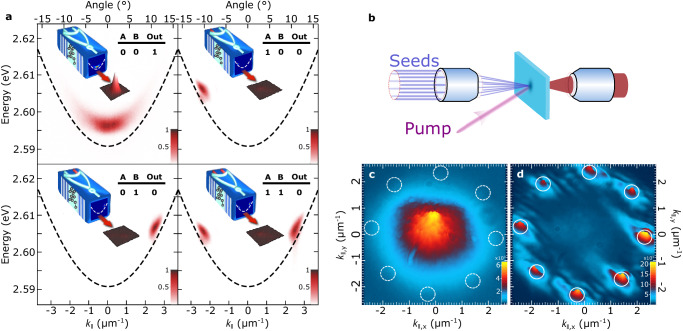
Universal polariton gate. **a** Normalized emission of the lower polariton branch in the four control input configurations annotated in each panel. The black dashed line is a parabolic fit to the lower polariton branch dispersion, illustrating the blue-shift of the polariton dispersion in the nonlinear regime. At the facet of each schematic we overlay the corresponding polariton dispersion image and the red arrows point to the corresponding spatial emission profile (~20 × 20 micrometers). **b** Schematic of the multi-input control-seed beams on a planar microcavity. The pump pulse injects a hot exciton reservoir, one molecular vibron-energy above the seeded states, and eight seed (control) pulses resonantly inject polaritons at equidistant in-plane wavevectors of the k-space ring of ∣k_∥_∣ ≈ 2.55 *μ*m^−1^. **c**, **d** Polariton emission in Fourier-space showing the switching of the gate between its logical “1” and logical “0” states. Color bars show relative intensity.

Figure 4 a shows the spatial profile of the emission intensity for the four input configurations of the two seed beams, and the corresponding dispersions demonstrating that in the presence of either of the inputs, non-ground-state amplification supersedes spontaneous ground-state condensation, resulting at the truth-table of the universal NOR gate, see inset of Fig. 3d. Utilizing the full two-dimensional parabolic polariton dispersion, we extend the NOR gate to a multi-input joint denial truth-functional operator. Using a spatial light modulator, we create 8 control pulses that are axisymmetric to *k*_∥_ = 0 and equidistant from each other across the k-ring. We focus all control beams on the same sample position at an incident angle of ~11^∘^ resonantly injecting into the system polaritons with an in-plane momentum of ∣k∣_∥_ ≈ 2.55 *μ*m^−1^ as shown schematically in Fig. 4b. By switching on any combination of the control-seed beams in the presence of the non-resonant pump beam, we can trigger the gate between its logical “1” and “0” states, depleting ground-state spontaneous polariton condensation by stimulating non-ground-state polariton amplification. Figure 4c, d show the measured emission intensity in reciprocal space in the absence and presence of all 8 seed beams respectively. We elaborate on the flexibility of our system by implementing various configurations in Section V in the SI and discuss a comparison with other optical inverters in Section VI in the SI.

**Fig. 5:**
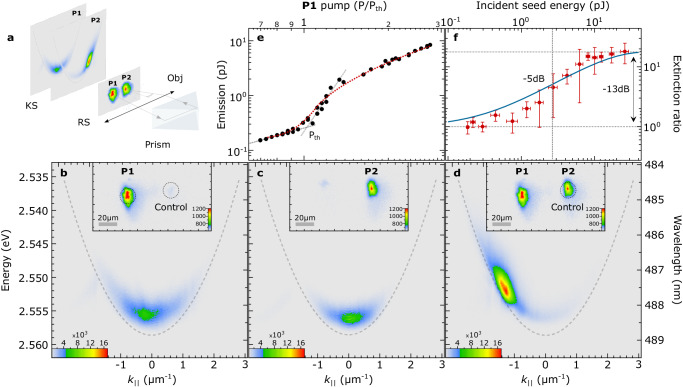
Cascadability of the NOT gate. **a** Experimental configuration for the proof-of-principle realization of cascading two consequent NOT gates. The polariton emission is shown in both real-space (RS) and spectrally resolved k-space (KS). **b** Time-averaged dispersion of ground-state polariton condensation from the first gate; the top inset shows the corresponding real-space emission under P1, with P = 2*P*_th_. Black dashed circle in the real-space inset indicates the position where redirected condensate emission resonantly excites the lower polariton dispersion of the second gate at an angle of ~6^∘^. **c** Time-averaged dispersion of ground-state polariton condensation from the second gate; the top inset shows the corresponding real-space emission under P2, with P = 2*P*_th_. The condensate emission spatially overlaps with the control beam from the first gate. **d** Time-averaged and spatially filtered dispersion of non-ground-state polariton amplification from the second gate temporarily overlapped with the control beam from the first gate; the top inset shows the corresponding real-space emission from the two gates, with P1 = P2 = 2*P*_th_. **e** Dependence of the polariton emission energy from the first gate on P1. **f** Extinction ratio of the ground-state polariton emission of the second NOT gate vs incident seed energy from the first gate; each red experimental data point represents a mean value derived from several measured points with standard deviation error bars, the blue curve shows the best fit of the numerical model and the gray vertical dashed line indicates the incident seed threshold energy for −5 dB extinction ratio between logic levels “1” and “0''. Color bars show the absolute emission intensity.

An essential aspect of logic gates is cascadability, i.e. the output of one gate to act as an input to another gate, a process that in optical gates requires degeneracy of the involved states. In the following, we investigate the cascadability of polariton negation gates operating on the principle of non-ground-state-amplification. Figure 5a illustrates the proof-of-concept configuration for cascading two NOT gates on the same ‘chip’ through out-of-cavity coupling. In this configuration, we use two spatially separated pump pulses, P1 & P2, at a distance that precludes in-plane coupling. In Fig. 5a we show the real-space and the spatially filtered dispersion from the two separate pump spots on the ‘chip’. The condensate emission of the first NOT gate (P1 output), acts as the control beam for the second gate, when redirected onto the ‘chip’ through a prism and overlapped spatially and temporally with the second pump, P2. In order to maximize the spectral overlap between the condensate emission and the non-ground-state lower polariton mode resonance, we redirect the emission at ~ 6^∘^. Smaller in-plane wavevectors for the control beam result in stronger ground-state condensate depletion, as also predicted theoretically and shown in the inset of Fig. 2a. Figure 5b, c show the nearly identical emission properties of the time-averaged dispersion images of the ground-state polariton condensates from the two gates when separately pumped at the gain saturation regime; 2*P*_th_. In the insets of Fig. 5b, c we show the transmitted time-averaged spatial emission of the two condensates. In the presence of P1, we note the spatial overlap of the polariton condensate emission in the region of the second gate. Figure 5d shows the spatially filtered dispersion of the second gate when the redirected polariton condensate emission of the first gate is temporally synchronized with P2. Evidently, the condensate control beam induces polariton amplification at a non-ground-state of the lower polariton dispersion of the second gate, whilst quenching the emission of condensate. We note here that in this instance the control beam energy is ≈4 pJ. Figure 5e shows the condensate emission energy vs P1. Figure 5f shows the extinction ratio of the condensate emission of the second gate vs the incident seed energy provided from the first condensate as derived from the calibration plot, red-dashed line, of Fig. 5e. We observe that the extinction ratio reaches a plateau at ≈−13 dB due to the exciton-reservoir depletion at the second gate. Assuming that an extinction ratio of −5 dB is the threshold for a workable NOT gate, it should be possible to cascade one gate to at least 2 other gates without regeneration of the output of the first gate. The blue solid line corresponds to the theoretical prediction. However, in cases where the output energy is not sufficient to drive multiple subsequent gates, or there is a necessity to achieve a higher contrast (depletion) of the ground-state emission, an additional signal regeneration stage mechanism can be implemented to increase the output energy, maximize the spectral overlap and, therefore, enable an improved on/off ratio and higher fan-out. For more information of the implementation of a regeneration stage please see Section VII of the SI.

By harnessing the concept of non-ground-state polariton amplification we realize a universal polariton NOR gate that provides the basic building block for a complete all-optical logic circuitry platform, operational at room temperature and high speed. Furthermore, the ability of a polariton NOR gate to operate with *N* inputs renders it equivalent to at least *N* − 1 conventional two-input transistors leading to a substantial decrease in the required transistor footprint. Future scale up to complex logic circuits will favorably make use of integrated planar architectures of photonic microcavities.

## Methods

### Sample fabrication

The sample is composed of a bottom distributed Bragg reflector (DBR), a central cavity defect region with an effective thickness slightly larger than half the exciton wavelength, and a top DBR on a fused silica substrate. The DBRs consist of alternating SiO_2_/Ta_2_O_5_ quarter-wavelength-thick layers produced by sputter deposition (9+0.5 pairs for the bottom DBR, 6+0.5 for the top DBR). The center of the cavity consists of a polymer layer sandwiched within 50-nm spacer layers of sputtered SiO_2_. The spacer is deposited on the organic using a SiO_2_ sputter target. The polymer, a methyl-substituted ladder-type poly(p-phenylene) (MeLPPP; *M*_*n*_ = 31500, *M*_*w*_ = 79000), is dissolved in toluene and spin-coated on the bottom spacer layer. The film thickness of approximately 35 nm is measured with a profilometer (Veeco Dektak).

### Spectroscopy

The pump pulse with ~200 fs duration is provided by a tunable optical parametric amplifier (Coherent OPerA SOLO) excited by 500 Hz high energy Ti:Sapphire regenerative amplifier (Coherent Libra-HE). The beam is spectrally filtered, providing 25 meV full-width at half-maximum (FWHM). The pulses are focused on the sample with a 50 mm lens at oblique incidence (45^∘^). The pump has an elliptical profile with ~40 *μ*m and 26 *μ*m spot sizes. The seed beams are produced by generating white light continuum (WLC) in a sapphire plate excited with 800 nm ultrashort (~100 fs) pulses. WLC is then spectrally filtered, resulting in broadband (2.59-2.7 eV) emission. The seed pulses are focused on the sample by an objective (10x Nikon, 0.3 NA), resulting in a spot size of ~25 *μ*m. A motorized translation stage with a retroreflector allows to adjust the temporal delay between the pump and seed pulses. For the complete experimental setup see SI, Section III.

Momentum- and energy-resolved emission is acquired in transmission configuration. Output emission of the sample is collected with an objective (10x Mitutoyo Plan Apo, NA = 0.28) and coupled to a spectrometer (Princeton Instruments SP2750) with an electron multiplying charge coupled device (EMCCD) camera (Princeton Instruments ProEM 1024BX3). The emission was spectrally resolved using a 1200 grooves/mm grating and a slit width of 200 *μ*m at the entrance of the spectrometer. An additional 1000 mm conjugated lens is used to project the Fourier plane of the collecting objective to the slit. The pump power dependence measurements (Fig. 2b, c) are obtained through integrating the output emission within ~*k*_∥_ ∈ (−2.96;−1.86) *μ*m^−1^ around k_probe_. The fluence of the seed beam is fixed at 45 nJ cm^−2^. To obtain the incident excitation density of the pump pulse, the average pump power is measured using a calibrated Si photodetector (Thorlabs-Det10/M) and an oscilloscope (Keysight DSOX3054T). Accuracy verification of the power measurements is carried out by using a powermeter: Si photodiode power sensor (Thorlabs-S120VC) with a console (Thorlabs-PM100D).

The seed power dependencies (Fig. 3a, d) are recorded with pumping at *P* ~ 2*P*_th_ for unseeded polariton condensation. Both seed beams have an incident angle of ~11^∘^ with the opposite momenta, e.g. *k*_∥,*B*_ = − *k*_∥,*A*_  ≈ − 2.55 *μ*m^−1^. The real space data depicting the NOR gate functionality (Fig. 4a) is obtained with angular filtering of the Fourier plane using an iris aperture.

### Multi-input NOR gate

WLC beam is generated by exciting a sapphire plate with 800 nm ultrashort (~100 fs) pulses, resulting in a broadband (2.59–2.75 eV) emission after spectral filtration with a combination of Semrock short-pass (FF01-492/SP-25) and long-pass (LP02-473RU-25) optical filters. A collimated beam of a Gaussian profile is then coupled to a visible range Spatial Light Modulator (SLM) that modified the phase front according to prepared phase masks of concentrically equidistant Gaussian spots in real-space. Real-space image of the first order SLM’s reflected phase plane is then conjugately projected on the objective (10x Nikon, 0.3 NA) aperture, which in turn focuses seed pulses on the sample in a single spot of ~25 *μ*m in diameter. The number of seed pulses and their incident angles are controlled by the phase mask on the SLM.

The seed amplification factor has been introduced in the following way: $SA=\frac{{P}_{2}-{P}_{1}}{{P}_{0}}$, where *P*_2_ is the power of the non-ground state condensate emission in the presence of the seed and the pump beams, *P*_1_ is the power of the output emission from the non-ground state area, where seeds would be applied, induced by the pump beam itself (without seeding non-ground state) and *P*_0_ is the power of the transmitted seeds in the absence of the pump beam.

### Supplementary information

Supplementary Information

Peer Review File

## Data Availability

The data that support the findings of this study are available from the corresponding author, P. G. Lagoudakis, upon request.
