# Supplementary material for: Room temperature, cascadable, all-optical polariton universal gates
Source: Nat Commun. 2024 Jun 25;15:5362. doi: 10.1038/s41467-024-49690-3 (PMC11199649; doi:10.1038/s41467-024-49690-3)
Supplement: Supplementary file 1 — Supplementary Information [file 41467_2024_49690_MOESM1_ESM.pdf]

Supplementary Information: Room temperature, cascadable, all-optical polariton universal gates

Denis A. Sannikov[1], Anton V. Baranikov[1], Anton D. Putintsev[1], Mikhail Misko[1], Anton V. Zasedatelev[1], Ullrich Scherf[2], and Pavlos G. Lagoudakis[1] [a]

[1]*Hybrid Photonics Laboratory, Skolkovo Institute of Science and Technology, Territory of Innovation Center Skolkovo, Bolshoy Boulevard 30, building 1, 121205 Moscow, Russia and*

[2]*Macromolecular Chemistry Group and Institute for Polymer Technology, Bergische Universität Wuppertal, Gauss-Strasse 20, 42119 Wuppertal, Germany*

(Dated: May 20, 2024)

[a] Correspondence address: p.lagoudakis@skol.tech

I. MICROSCOPIC MODEL OF THE SYSTEM. NUMERICAL SIMULATIONS

In this work we use numerical simulations to investigate the effect of ground state condensate depletion, when a small seed pulse is applied at a non-zero angle at the sample together with the pumping pulse. We modify a microscopic model used to describe the dynamics of the system in [1, 2] and solve a master equation for the density matrix of lower branch polaritons subsystem:

$$\frac{d\hat{\rho}}{dt} = \frac{i}{\hbar}\left[\hat{\rho}, \hat{H}_T\right] + \hat{L}_T(\hat{\rho}) \tag{1}$$

Here, the hermitian part consists of the light-matter Jaynes Cummings Hamiltonian and a standard optomechanical Fröhlich Hamiltonian for vibron-exciton interactions: $\hat{H}_T = \hat{H}_{JC} + \hat{H}_{vib,exc-vib}$. The Lindblad terms include dissipation of lower polaritons $\hat{L}_{low}(\hat{\rho})$, hot excitons $\hat{L}_{hot}(\hat{\rho})$, vibrons $\hat{L}_{vib}(\hat{\rho})$. Also we take into account non-resonant pumping and seeding – $\hat{L}_{pump}(\hat{\rho})$ and $\hat{L}_{seed}(\hat{\rho})$ respectively. The intrabranch thermalization processes are included as $\hat{L}_{therm}(\hat{\rho})$.

In this section we one by one describe all these terms in detail and derive the system of differential equations that describe the dynamics of polariton populations of the LPB $n_{low\mathbf{k}}(t)$.

Light-Matter interactions. Strong Coupling

We start by introducing the creation and annihilation operators for the microcavity photonic modes that obey a bosonic commutation relation:

$$\left[\hat{a}_{cav\mathbf{k}}, \hat{a}^{+}_{cav\mathbf{k'}}\right] = \delta_{\mathbf{kk'}} \tag{2}$$

Each mode has an in-plane momentum $\mathbf{k}$ and an energy $\hbar\omega_{cav\mathbf{k}}$. The dispersion is parabolic with a cut-off frequency $\omega_{cav0} = 2.64$ eV and a curvature parameter $\alpha_{cav} = 2.2$ meV $\cdot$ µm^2: $\omega_{cav\mathbf{k}} = \omega_{cav0} + \alpha_{cav}|\mathbf{k}|^2$.

The collective electronic excitations of the ensemble of organic molecules are also described with the formalism of creation and annihilation operators in the k-space. Excitons are known to be dispersionless quasi-particles, $\omega_{exc\mathbf{k}} = 2.72$ eV. Since we assume that the chance to find a segment of the polymer already in an excited state is low, the bosonic

commutation relation is valid for these operators.

$$\left[\hat{c}_{exc\mathbf{k}}, \hat{c}^{+}_{exc\mathbf{k'}}\right] = \delta_{\mathbf{kk'}} \tag{3}$$

The standard Jaynes-Cummings Hamiltonian of electronic transitions in this ensemble of organic molecules coupled to the microcavity photonic modes with an in-plane momentum looks as follows:

$$\hat{H}_{JC} = \sum_{\mathbf{k}} \hbar\omega_{cav\mathbf{k}} \hat{a}^{+}_{cav\mathbf{k}} \hat{a}_{cav\mathbf{k}} + \sum_{\mathbf{k}} \hbar\omega_{exc\mathbf{k}} \hat{c}^{+}_{exc\mathbf{k}} \hat{c}_{exc\mathbf{k}} + \sum_{\mathbf{k}} \hbar\Omega_R \left(\hat{c}^{+}_{exc\mathbf{k}} \hat{a}_{cav\mathbf{k}} + \hat{c}_{exc\mathbf{k}} \hat{a}^{+}_{cav\mathbf{k}}\right) \tag{4}$$

In the considered case the Rabi frequency $\Omega_R \approx 85$ meV is higher than the exciton losses rate $\gamma_{exc} \approx 0.01$ meV (corresponds to the 40 ps exponential decay, observed in [3]) and photon loss rate $\gamma_{cav} = 4.4$ meV, meaning that the system operates in a strong coupling regime. We diagonalize the Hamiltonian by performing a unitary transformation of operators:

$$\hat{s}_{low\mathbf{k}} = \hat{a}_{cav\mathbf{k}} \cos\phi_{\mathbf{k}} - \hat{c}_{exc\mathbf{k}} \sin\phi_{\mathbf{k}} \tag{5}$$

$$\hat{s}_{up\mathbf{k}} = \hat{a}_{cav\mathbf{k}} \sin\phi_{\mathbf{k}} + \hat{c}_{exc\mathbf{k}} \cos\phi_{\mathbf{k}} \tag{6}$$

After the procedure we obtain the lower and upper polariton branches (LPB and UPB respectively) shown in Fig.S1. Grey dashed lines represent the uncoupled exciton mode (horizontal) and cavity dispersion (parabolic), and color of the branches correspond to the photonic fraction of the polaritons. The analytical expressions for UPB and LPB are the following:

$$\omega_{low\mathbf{k},up\mathbf{k}} = \frac{\omega_{exc} + \omega_{cav\mathbf{k}}}{2} \mp \sqrt{\frac{(\omega_{exc} - \omega_{cav\mathbf{k}})^2}{4} + \Omega^2_{R\mathbf{k}}} \tag{7}$$

Vibron-assisted transitions. Fröhlich Hamiltonian

The main mechanism of exciton relaxations is the interaction with the vibron subsystem of the molecule ensemble. It has been demonstrated in Ref.[4] that resonant vibron transitions can decrease the condensation threshold as much as 10 times and allow for extremely efficient switching at low energies.

All experiments were carried out such that the lower polariton modes were exactly one vibron quantum below the pumped reservoir of hot excitons allowing efficient resonant single

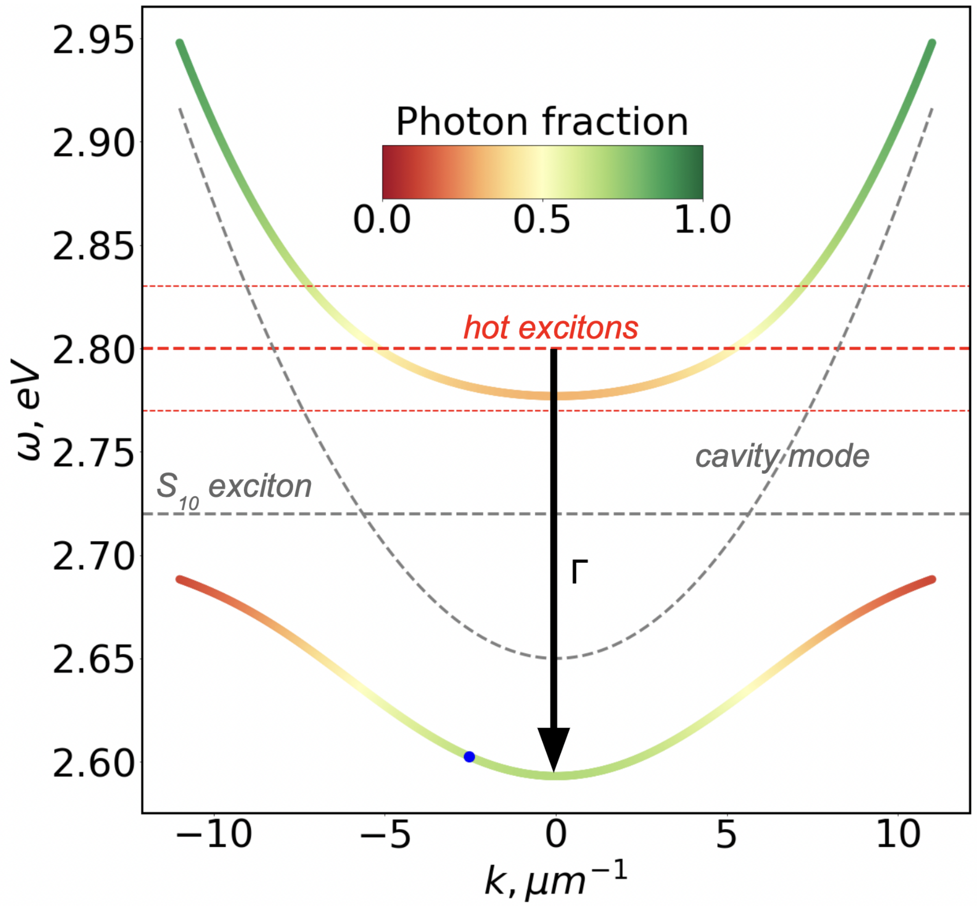

FIG. S1. **Dispersion of the system.** Color, i.e. "color scale", represents the absolute square of the photonic Hopfield coefficient for both UPB and LPB. The state where the resonant seed is applied in the experiment is marked with the blue dot. The hot exciton reservoir is depicted with a thick dashed red line, and two thin dashed red lines indicate a FWHM of its inhomogeneous broadening. The solid black arrow represents the average transition rate from the reservoir to the LPB modes.

vibron transitions. To describe this configuration theoretically we introduce the Fröhlich type optomechanical Hamiltonian of collective exciton and collective vibron interactions in the momentum space with a coupling constant g:

$$\hat{H}_{vib,exc-vib} = \sum_{\mathbf{q}} \hbar\omega_{vib}\hat{b}^{+}_{\mathbf{q}}\hat{b}_{\mathbf{q}} + \sum_{\mathbf{k}}\sum_{\mathbf{k'}} \hbar g \hat{c}^{+}_{exc\mathbf{k}}\hat{c}_{exc\mathbf{k'}} \left(\hat{b}^{+}_{-(\mathbf{k}-\mathbf{k'})} + \hat{b}_{\mathbf{k}-\mathbf{k'}}\right) \tag{8}$$

Here, $\hat{b}^{+}_{\mathbf{q}}$ and $\hat{b}_{\mathbf{q}}$ are bosonic creation and annihilation for vibrons with the momentum $\mathbf{q}$ respectively. We then rewrite this Hamiltonian in the new polariton basis, noting that in the particular experimental configuration, the pump is applied non-resonantly to hot excitons

with higher energy (2.8 eV) that are uncoupled to the cavity modes, so the corresponding excitonic fraction in the transformed polaritonic coordinates for the pumped mode is one and the photonic fraction is zero.

$$\hat{H} = \sum_{\mathbf{k}} \hbar\omega_{low\mathbf{k}} \hat{s}^{+}_{low\mathbf{k}} \hat{s}_{low\mathbf{k}} + \sum_{\mathbf{k}} \hbar\omega_{up\mathbf{k}} \hat{s}^{+}_{up\mathbf{k}} \hat{s}_{up\mathbf{k}} + \sum_{\mathbf{q}} \hbar\omega_{vib} \hat{b}^{+}_{\mathbf{q}} \hat{b}_{\mathbf{q}} + \sum_{\mathbf{k}} \sum_{\mathbf{k'}} \hbar g \left(\hat{s}^{+}_{up\mathbf{k}} \cos\phi_{\mathbf{k}} - \hat{s}^{+}_{low\mathbf{k}} \sin\phi_{\mathbf{k}} \right) \left(\hat{s}_{up\mathbf{k'}} \cos\phi_{\mathbf{k'}} - \hat{s}_{low\mathbf{k'}} \sin\phi_{\mathbf{k'}} \right) \left(\hat{b}^{+}_{-(\mathbf{k}-\mathbf{k'})} + \hat{b}_{\mathbf{k}-\mathbf{k'}} \right) \tag{9}$$

The upper polariton branch is not pumped at any time, as we choose the in plane momentum of the pumping beam $k = 10$ μm^{-1}. It is visible from Fig.S1 that at this k the energy of the UPB is much higher than the pump (2.8 eV). Therefore, we will only consider transitions from the uncoupled hot excitons ($\left[\hat{c}_{hot\mathbf{k}}, \hat{c}^{+}_{hot\mathbf{k'}}\right] = \delta_{\mathbf{k}\mathbf{k'}}$) to the lower polariton branch that happen with the creation of a vibron. It is assumed that the thermal number of vibrons is negligible at room temperature since $\hbar\omega_{vib} \gg T$; $n_{vib\mathbf{q}} = \langle \hat{b}^{+}_{\mathbf{q}} \hat{b}_{\mathbf{q}} \rangle \ll 1$

Thermalization. Lindblad equations.

Since the system is non-isolated, all the considered quasi-particles interact with the environment, resulting in losses. In our model we describe all relaxation processes with Lindblad superoperators that act on the total "polariton+vibron+uncoupled excitons" system density matrix $\hat{\rho}$. The superoperators that describe the relaxations of lower polaritons and the hot exciton reservoir are:

$$\begin{aligned} \hat{L}_{low}(\hat{\rho}) &= \sum_{\mathbf{k}} \frac{\gamma_{low\mathbf{k}}}{2} \left(2\hat{s}_{low\mathbf{k}} \hat{\rho} \hat{s}^{+}_{low\mathbf{k}} - \hat{s}^{+}_{low\mathbf{k}} \hat{s}_{low\mathbf{k}} \hat{\rho} - \hat{\rho} \hat{s}^{+}_{low\mathbf{k}} \hat{s}_{low\mathbf{k}} \right) \\ \hat{L}_{hot}(\hat{\rho}) &= \sum_{\mathbf{k}} \frac{\gamma_{hot}}{2} \left(2\hat{c}_{hot\mathbf{k}} \hat{\rho} \hat{c}^{+}_{hot\mathbf{k}} - \hat{c}^{+}_{hot\mathbf{k}} \hat{c}_{hot\mathbf{k}} \hat{\rho} - \hat{\rho} \hat{c}^{+}_{hot\mathbf{k}} \hat{c}_{hot\mathbf{k}} \right) \end{aligned} \tag{10}$$

Here, $\gamma_{low\mathbf{k}} = \gamma_{cav} \cos^2\phi_{\mathbf{k}} + \gamma_{exc} \sin^2\phi_{\mathbf{k}}$ and γ_{hot} corresponds to the 200 fs effective cooling time of the hot excitons [5]. We consider the number of excited vibrons to be negligible. Under this assumption the Lindbladian for vibrons is similar:

$$\hat{L}_{vib}(\hat{\rho}) = \sum_{\mathbf{q}} \frac{\gamma_{vib}}{2} \left(2\hat{b}_{\mathbf{k}} \hat{\rho} \hat{b}^{+}_{\mathbf{q}} - \hat{b}^{+}_{\mathbf{q}} \hat{b}_{\mathbf{q}} \hat{\rho} - \hat{\rho} \hat{b}^{+}_{\mathbf{q}} \hat{b}_{\mathbf{q}} \right) \tag{11}$$

The single vibron transitions were described in the hermitian part of the equation. In order to describe thermalization processes that happen within the bottom of the lower polariton

branch by means of interactions with the thermal bath of low energy molecular vibrations we introduce the corresponding Lindblad superoperator:

$$\hat{L}_{therm}(\hat{\rho}) = \sum_{\mathbf{k}_1,\mathbf{k}_2} \frac{\gamma_{therm}^{\mathbf{k}_2\to\mathbf{k}_1}}{2}(2\hat{s}_{low\mathbf{k}_2}\hat{s}^{+}_{low\mathbf{k}_1}\hat{\rho}\hat{s}_{low\mathbf{k}_1}\hat{s}^{+}_{low\mathbf{k}_2} - \\ -\hat{s}_{low\mathbf{k}_1}\hat{s}^{+}_{low\mathbf{k}_2}\hat{s}_{low\mathbf{k}_2}\hat{s}^{+}_{low\mathbf{k}_1}\hat{\rho} - \hat{\rho}\hat{s}_{low\mathbf{k}_1}\hat{s}^{+}_{low\mathbf{k}_2}\hat{s}_{low\mathbf{k}_2}\hat{s}^{+}_{low\mathbf{k}_1}) \tag{12}$$

Here, we denoted the transition rate of polaritons from LPB mode with the in-plane momentum $\mathbf{k}_1$ to the mode with $\mathbf{k}_2$ as $\gamma_{therm}^{\mathbf{k}_1\to\mathbf{k}_2}$. We assume the downward transition rates to be k-independent: $\gamma_{therm}^{\mathbf{k}_1\to\mathbf{k}_2} = \gamma_{therm}, \quad \omega(\mathbf{k}_1) > \omega(\mathbf{k}_2)$. However, when interacting with thermal bath, the inverse transition rate obeys the Kubo-Martin-Schwinger relation:

$$\gamma_{therm}^{\mathbf{k}_2\to\mathbf{k}_1} = \gamma_{therm}^{\mathbf{k}_1\to\mathbf{k}_2} \exp\left\{\left(\frac{\hbar\omega_2 - \hbar\omega_1}{k_B T}\right)\right\} \tag{13}$$

This effectively means that thermalization processes dissipate the energy and upward transitions are less possible within the lower polariton branch.

We introduce the pumping of the hot excitons and resonant seeding of lower polaritons by means of similar Lindblad operators:

$$\hat{L}_{pump}(\hat{\rho}) = +\sum_{\mathbf{k}} \frac{\kappa_{p\mathbf{k}}(t)}{2}\left(2\hat{c}_{hot\mathbf{k}}\hat{\rho}\hat{c}^{+}_{hot\mathbf{k}} - \hat{c}^{+}_{hot\mathbf{k}}\hat{c}_{hot\mathbf{k}}\hat{\rho} - \hat{\rho}\hat{c}^{+}_{hot\mathbf{k}}\hat{c}_{hot\mathbf{k}}\right) + \\ +\sum_{\mathbf{k}} \frac{\kappa_{p\mathbf{k}}(t)}{2}\left(2\hat{c}^{+}_{hot\mathbf{k}}\hat{\rho}\hat{c}_{hot\mathbf{k}} - \hat{c}_{hot\mathbf{k}}\hat{c}^{+}_{hot\mathbf{k}}\hat{\rho} - \hat{\rho}\hat{c}_{hot\mathbf{k}}\hat{c}^{+}_{hot\mathbf{k}}\right) \tag{14}$$

$$\hat{L}_{seed}(\hat{\rho}) = \sum_{\mathbf{k}} \frac{\kappa_{seed\mathbf{k}}(t)}{2}\left(2\hat{s}_{low\mathbf{k}}\hat{\rho}\hat{s}^{+}_{low\mathbf{k}} - \hat{s}^{+}_{low\mathbf{k}}\hat{s}_{low\mathbf{k}}\hat{\rho} - \hat{\rho}\hat{s}^{+}_{low\mathbf{k}}\hat{s}_{low\mathbf{k}}\right) + \\ +\sum_{\mathbf{k}} \frac{\kappa_{seed\mathbf{k}}(t)}{2}\left(2\hat{s}^{+}_{low\mathbf{k}}\hat{\rho}\hat{s}_{low\mathbf{k}} - \hat{s}_{low\mathbf{k}}\hat{s}^{+}_{low\mathbf{k}}\hat{\rho} - \hat{\rho}\hat{s}_{low\mathbf{k}}\hat{s}^{+}_{low\mathbf{k}}\right) \tag{15}$$

We consider pump and seed rates to be gaussian in the time domain with a fixed FWHM:

$$\kappa_{p\mathbf{k}}(t) = P\exp\left\{\left(-\frac{(t-t_p^{arr})^2}{\tau_p^2}\right)\right\}\delta(k-k_{pump}) \\ \kappa_{seed\mathbf{k}}(t) = S\exp\left\{\left(-\frac{(t-t_{seed}^{arr})^2}{\tau_{seed}^2}\right)\right\}\exp\left\{\left(-\frac{(k-k_{seed})^2}{\sigma_{seed}^2}\right)\right\} \tag{16}$$

Here the full widths at half maximum of the pulses are $\tau_{p,seed}^{FWHM} = 1.665\tau_{p,seed}$. The arrival times of pump and seed are denoted as t_p^{arr} and t_{seed}^{arr} respectively, P and S are the intensity parameters of the beams, σ_{seed} is the angular width of the seed pulse as we consider it to be Gaussian in k-domain as well.

Rate equations

After the hermitian and the non-hermitian parts are ready, we finally write the Lindblad master equation for the full polariton+vibron density matrix:

$$\frac{d\hat{\rho}}{dt} = \frac{i}{\hbar}\left[\hat{\rho}, \hat{H}\right] + \hat{L}_{hot}(\hat{\rho}) + \hat{L}_{low}(\hat{\rho}) + \hat{L}_{vib}(\hat{\rho}) + \hat{L}_{therm}(\hat{\rho}) + \hat{L}_{pump}(\hat{\rho}) + \hat{L}_{seed}(\hat{\rho}) \tag{17}$$

In order to find the dynamics of particle numbers in modes on the lower and upper polariton branches we trace equation Eq.17 with the corresponding particle number operators:

$$\frac{dn_{low\mathbf{k}}}{dt} = Tr\left(\hat{s}^{+}_{low\mathbf{k}}\hat{s}_{low\mathbf{k}}\dot{\rho}\right) \tag{18}$$

While tracing, three particle averages will inevitably occur due to the nature of Fröhlich Hamiltonian, which correspond to the annihilation of a hot exciton, and creation of lower polariton and a vibron. Since the vibron quality factor is much smaller than the cavity quality factor [1] we can exclude the vibron subsystem adiabatically, arriving at the following continuous set of equations for the polariton and hot exciton subsystem:

$$\begin{aligned}\frac{dn_{hot\mathbf{k}}}{dt} &= -\gamma_{hot\mathbf{k}}n_{hot\mathbf{k}} + \kappa_{hot\mathbf{k}}(t) - \sum_{\mathbf{k'}}\Gamma^{hot\mathbf{k}}_{low\mathbf{k'}}n_{hot\mathbf{k}}(n_{low\mathbf{k'}}+1)\\ \frac{dn_{low\mathbf{k}}}{dt} &= -\gamma_{low\mathbf{k}}n_{low\mathbf{k}} + \kappa_{seed\mathbf{k}}(t) + \sum_{\mathbf{k'}}\Gamma^{hot\mathbf{k'}}_{low\mathbf{k}}n_{hot\mathbf{k'}}(n_{low\mathbf{k}}+1)+\\ &+\sum_{\mathbf{k'}}\left[\gamma^{\mathbf{k'}\to\mathbf{k}}_{therm}n_{low\mathbf{k'}}(n_{low\mathbf{k}}+1) - \gamma^{\mathbf{k}\to\mathbf{k'}}_{therm}n_{low\mathbf{k}}(n_{low\mathbf{k'}}+1)\right]\end{aligned} \tag{19}$$

The vibron assisted resonant transition rates Γ^{hot}_{low} from the hot exciton reservoir to LPB modes in the adiabatic approximation can be described as:

$$\Gamma^{hot}_{low} = \frac{g^2 X_{low}(\gamma_{low}+\gamma_{hot}+\gamma_{vib})}{(\omega_{hot}-\omega_{low}-\omega_{vib})^2 + \frac{1}{4}(\gamma_{low}+\gamma_{hot}+\gamma_{vib})^2} \tag{20}$$

Here X_{low} is the exciton fractions of the corresponding LPB mode. We notice however that due to the inhomogeneous broadening of the hot exciton reservoir the vibron assisted transitions are almost uniformly effective within the bottom of LPB (see Fig.S1). We use Eq.20 to calculate the uniform effective transition rate Γ assuming the spectral profile of the inhomogeneously broadened reservoir to be gaussian with FWHM of 60meV. $\Gamma = \int \Gamma^{hot}_{low}\rho(\omega_{hot})d\omega_{hot} \approx 3\times 10^{-6}$ eV

In order to obtain the system with a finite number of equations and solve it numerically we need to discretize the system. We consider a set of modes at the bottom of LPB equidistant

in frequency domain. Such discretization leads to a uniform density of states, since we are working with a 2D system with parabolic dispersion. In such case the density of states for the sample under consideration is according to [1]:

$$D_{\omega_j} = \begin{cases} 10^5\delta\omega, & \omega_j > \omega_{low}(0) \\ 1, & \omega_j = \omega_{low}(0) \end{cases} \tag{21}$$

Now we can rewrite Eq.17 for the discrete set of modes at the bottom of LPB n_i and the pumped mode $n_P \equiv n_{hot}$. Since the energy difference between the LPB modes within the region of interest is small (15 meV at max) we consider the downwards thermalization transitions to be aslo k-independent: $\gamma_{therm}^{\mathbf{k}_1\to\mathbf{k}_2} = \gamma_{therm}, \quad \omega_1 > \omega_2$. The inverse transitions obey Kubo-Martin-Schwinger relations: $\gamma_{therm}^{\mathbf{k}_1\to\mathbf{k}_2} = \gamma_{therm}\exp\{(-\hbar(\omega_2-\omega_1)/k_BT)\}, \quad \omega_1 < \omega_2$. The discrete set of equations in this case is:

$$\begin{aligned} \frac{dn_P}{dt} &= -\gamma_P n_P + \kappa_P(t) - \sum_j \Gamma n_P(n_j + D_j) \\ \frac{dn_i}{dt} &= -\gamma_i n_i + \kappa_{seed}(\mathbf{k}_i, t) + \Gamma n_P(n_i + D_i) + \\ &+ \sum_j \left[\gamma_{therm}^{j\to i} n_j(n_i + D_i) - \gamma_{therm}^{i\to j} n_i(n_j + D_j)\right] \end{aligned} \tag{22}$$

We numerically solve the system of Eqs.22 for $N = 31$ modes of LPB including the ground state mode for various model parameters. In order to compare the results of the simulations with experiment, we need to investigate not only the sub-picosecond dynamics of the polariton occupancies, but also measure time-integral quantities. The most frequently used parameter throughout this work is the total integral emission that was registered by a spectrometer at a specific angle:

$$I_{|k_\parallel|<k_{max}} = \int_0^\infty dt \sum_{i:|\mathbf{k}_i|<k_{max}} \frac{\gamma_i}{D_i} n_i(t)\Delta k_i \tag{23}$$

II. GROUND-STATE CONDENSATE DEPLETION

In this section we investigate the parameter space of the system. The quantity, that is represented on the colour map of Fig.S2, which is an enlarged plot of the inset in Fig.2a in the main manuscript, and acts as a successful negation operation marker, is the depletion of the ground state condensate $D = \log_{10} I/I_0$, where I_0 is the emission integrated over time

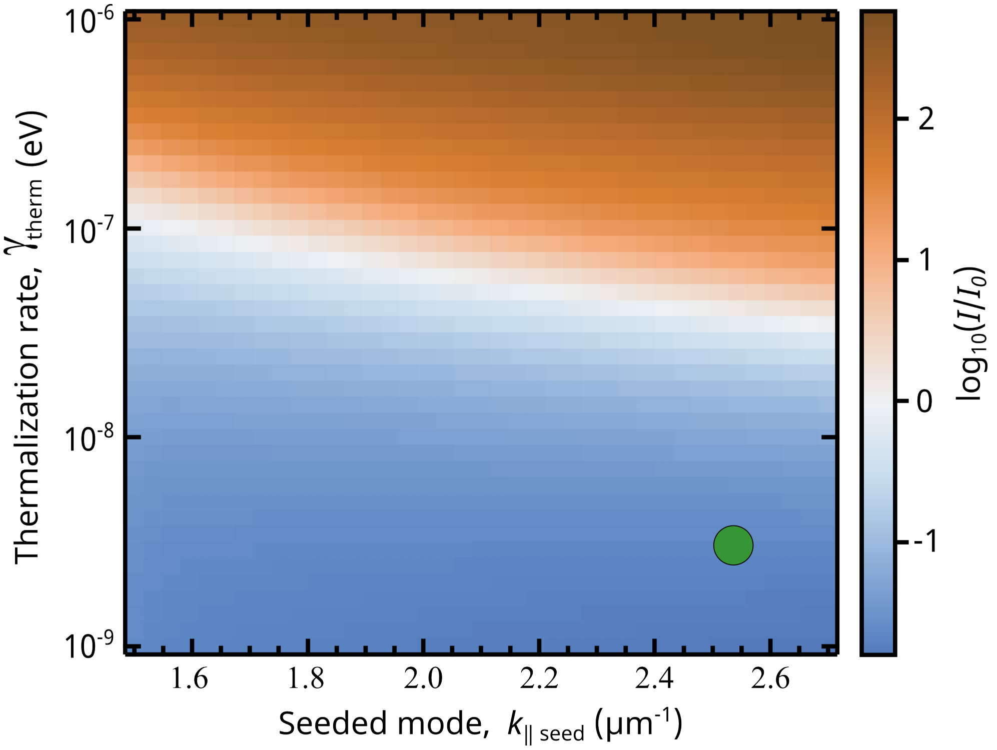

FIG. S2. **Parameter space of non-ground-state amplification.** Amplification (in orange)/depletion (in blue) of the ground state condensate. Pumping pulse energy is fixed at $2P_{\rm th}$. The green dot marks the best fit parameters (see Fig.3 in the main text). This figure is an enlarged plot of the inset of Fig.2a in the main manuscript.

and over $|\mathbf{k}_{\parallel}| < 1\ \mu\mathrm{m}^{-1}$ from the cavity in the presence of the pumping beam only, and I is the same quantity obtained in the presence of a seeding beam at $\mathbf{k}_{\parallel} \neq 0$. Blue areas correspond to $D < 0$, which means that the condensate is depleted, and therefore a NOT/NOR operation can be performed. Orange areas correspond to cases when amplification of the ground state condensate happens instead.

Figure S2 represents simulations results for a series of thermalization constants γ_{therm} and seeded mode positions. All the simulations are conducted at pumping rate $P = 2P_{\rm th}$. Thermalization processes between the ground state and the seeded state are the key restricting factor to the depletion of the ground-state condensate. In case of high thermalization constant, the preoccupation in the seeded mode will act as an additional pump source, resulting in amplification of the ground state condensate (orange areas) rather than desired depletion. In the formal absence of thermalization ($\gamma_{therm} = 0$), the depletion will formally

happen even below the condensation threshold. The thermalization rate that corresponds to the best fit of both dependencies in Figs.3a,b of the main text is around $[0.3 - 0.4] \times 10^{-8}$ eV. The corresponding best fit point in the parameter space is marked with a green dot.

The dependence of the ground state depletion on the frequency of the selected mode is explained by Kubo-Martin-Schwinger relation: in case when the frequency difference between the excited mode and the ground state is of the same order or higher as the temperature (which in our case is ambient) the inverse transitions from the ground state to the excited state become exponentially less effective resulting in worse depletion of the ground state.

III. THE EXPERIMENTAL SETUP

All the experiments described in the main text have been carried out using the following experimental arrangement. To realise NOT and NOR gates operation at room temperature, we configure non-ground state dynamical polariton amplification. As seed beams we employ a white-light continuum (WLC) generated in a sapphire plate split into two beams with equal intensity, the seed beams "A" and "B". Afterwards, both the seed beams are focused on the same spot by the same objective. To access non-ground polariton states within the lower polariton branch, we seed the system under a different angle of incidence that we control through the translation stages in optical paths of both seed beams accordingly. To control the temporal overlap between the pump and both seed beams, we utilise two motorized delay lines - translation stages equipped with retroreflectors, as shown in Fig.S3. The output emission is filtered in the Fourier plane of the objective by a home-made short pass $k_{\parallel}$-filter, which cuts the light with in-plane momentum $|\mathbf{k}_{\parallel}| > 1$ μm^{-1}.

We have filtered the WLC beam spectrally by a short pass filter to avoid seeding the lower-lying polariton states through possible leaky modes and sample defects (in particular states around $\mathbf{k}_{\parallel} = 0$). Figure S4 shows the spectrum of the seed beams with respect to typical energy bands of the ground state as well as non-zero momentum polariton states under investigation depicting the complete suppression of parasitic scattering from the seed beams in the vicinity to the ground polariton state at $\mathbf{k}_{\parallel} = 0$ (2.59 eV).

We further extend the functionality of the polariton NOR gate by investigating the tolerance of the effect with respect to the angle of incidence for the seed beams. We measure the extinction for the NOR gate, i.e. the ratio between high and low level output, expressed in

dB versus in-plane momentum of resonantly injected polaritons by one of the seed beams. Figure S5 shows the flexibility of the gate under the broad range of seeded states, which allows for tuning the energy of the seed beams. As we expect, extinction gradually decreases with increasing $k_{\|}$ (the bottom x-axis) as we lose the resonance with the vibron for higher energy polariton states.

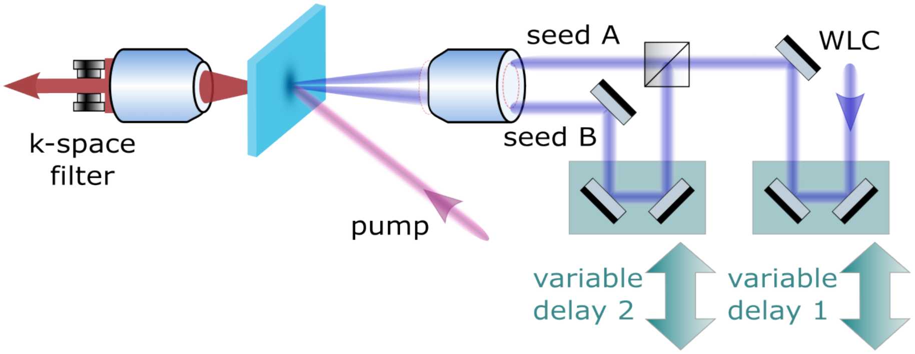

FIG. S3. **Scheme of the experiment.** Filtered WLC is split into two beams, seed A and B. We use variable delay lines in each arm to maximize temporal overlap between pump and both seed beams.

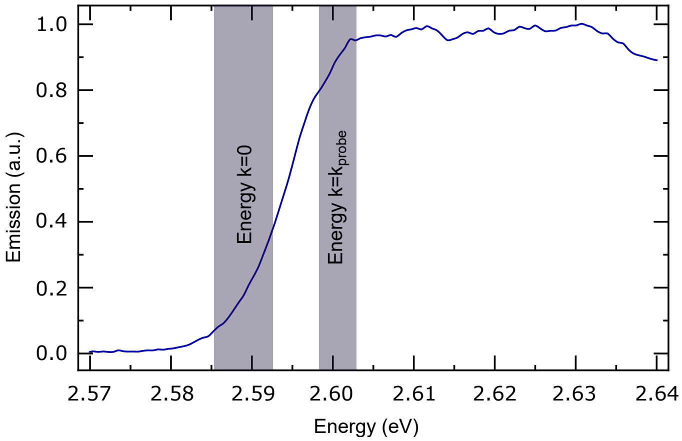

FIG. S4. **The seed spectrum.** The spectrum of seed beams (blue) after passing short pass filters that cut out low energy photons to prevent for seed scattering at the energy corresponding to $\mathbf{k}_{\|} = 0$. Gray-shaded areas show typical energy bands of the ground state as well as non-zero momentum polariton states under investigation.

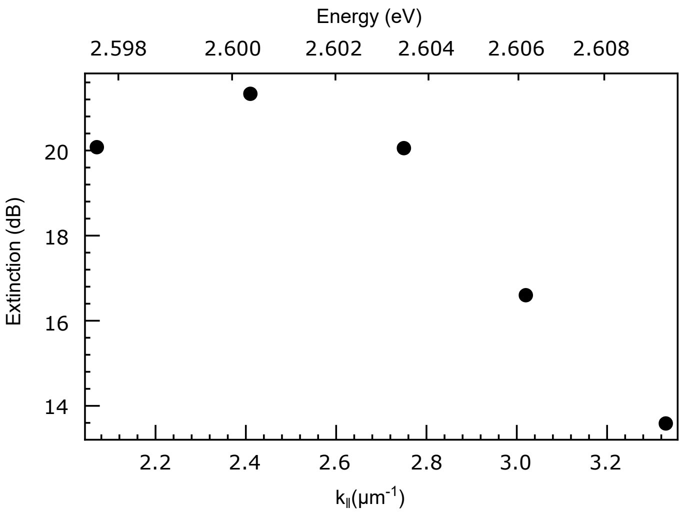

FIG. S5. **Flexibility of the polariton NOR gate.** The gate operates at room-temperature within the broad range of seeded states, which allows for tuning the energy of seed beams. The extinction gradually decreases with increasing $k_{\|}$ (the bottom x-axis) as we lose the resonance with vibron at higher energy polariton states. The top x-axis shows the energy of the corresponding polaritons states resonantly injected by the seed beam.

IV. UNSEEDED POLARITON CONDENSATION

Intense vibronic resonances of MeLPPP make polariton condensation easy even without any prearrangement in polariton occupancy. Relying on the single-step vibron-mediated exciton-to-polariton relaxation process, we realise unseeded polariton condensation that we harness in our binary logic as the high level - "1". Figure S6a shows a schematic of the optical excitation that forms a hot exciton reservoir, which is superimposed with the dispersion relation of the polariton system under investigation. Note the energy of hot excitons (2.8 eV) here is one vibronic energy quantum (0.2 eV) above the ground polariton state (2.6 eV). Energy-momentum distributions of emission recorded below ($P \sim 100\ \mu\mathrm{J} \cdot \mathrm{cm}^{-2}$) and above ($P \sim 280\ \mu\mathrm{J} \cdot \mathrm{cm}^{-2}$) the threshold are shown in Fig.S6b as the top and bottom panels respectively. Analysis of the emission integrated over $k_{\|} = 0 \pm 1.6\ \mu\mathrm{m}^{-1}$ clearly shows superlinear rise of polariton occupancy above the threshold of $\sim 230\ \mu\mathrm{J} \cdot \mathrm{cm}^{-2}$ (top panel in Fig.S6c) accompanied with significant line narrowing and blueshift (bottom panel in Fig.S6c) - commonly recognized features of polariton condensation in organic microcavities.

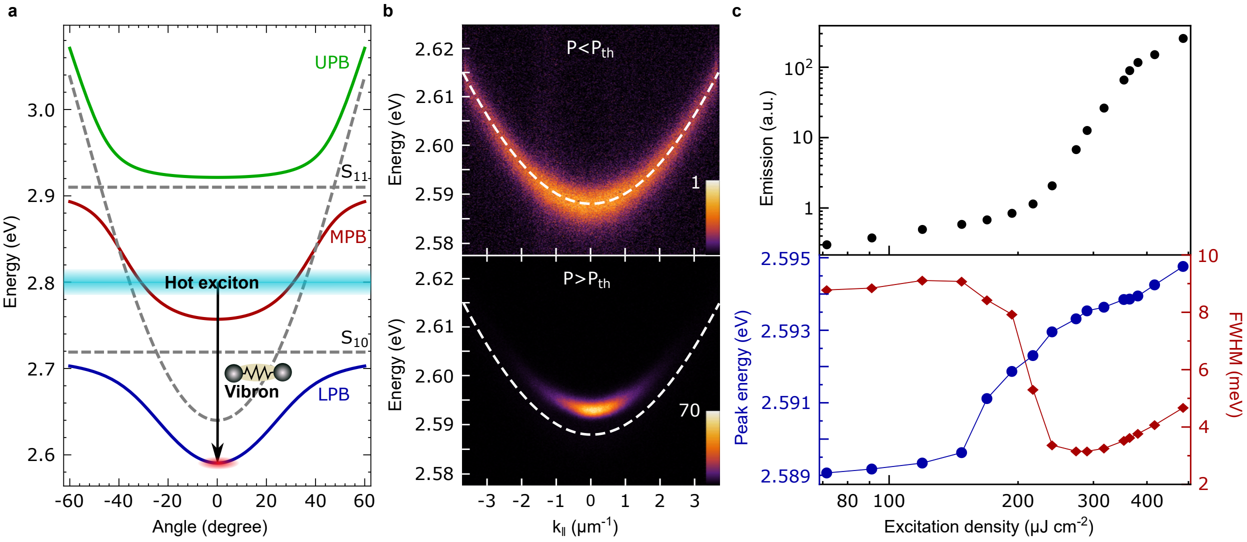

FIG. S6. **Unseeded polariton condensation.** **a**, Schematic of the optical excitation (hot excitons) superimposed with upper/middle/lower polariton branches drawn in green, red, and blue solid lines, respectively. Parabolic-dashed line demonstrates the bare cavity mode, and two horizontal-dashed lines show two sub-levels of the first excited singlet state of MeLPPP (S_{10} and S_{11}). The optical pump is aligned at 45 degrees incidence with 2.8 eV photon energy effectively injecting "hot" excitons depicted with a blue-shaded area. The vibron-mediated hot exciton-to-polariton relaxation process is depicted by a black solid vertical arrow. **b**, The top and the bottom panels show E, k-distribution of the emission below ($P \sim 100\ \mu\text{J} \cdot \text{cm}^{-2}$) and above ($P \sim 280\ \mu\text{J} \cdot \text{cm}^{-2}$) the condensation threshold respectively, where the dashed line corresponds to the unperturbed lower polariton branch. **c**, The top panel depicts the output emission integrated over $k_{\parallel} = 0 \pm 1.6\ \mu\text{m}^{-1}$ versus excitation density, while the bottom panel shows the spectral characteristics of the emission, namely its peak position (blue) and the full-width half-maximum (FWHM, red).

V. MULTI-INPUT NOR GATE CONFIGURATIONS

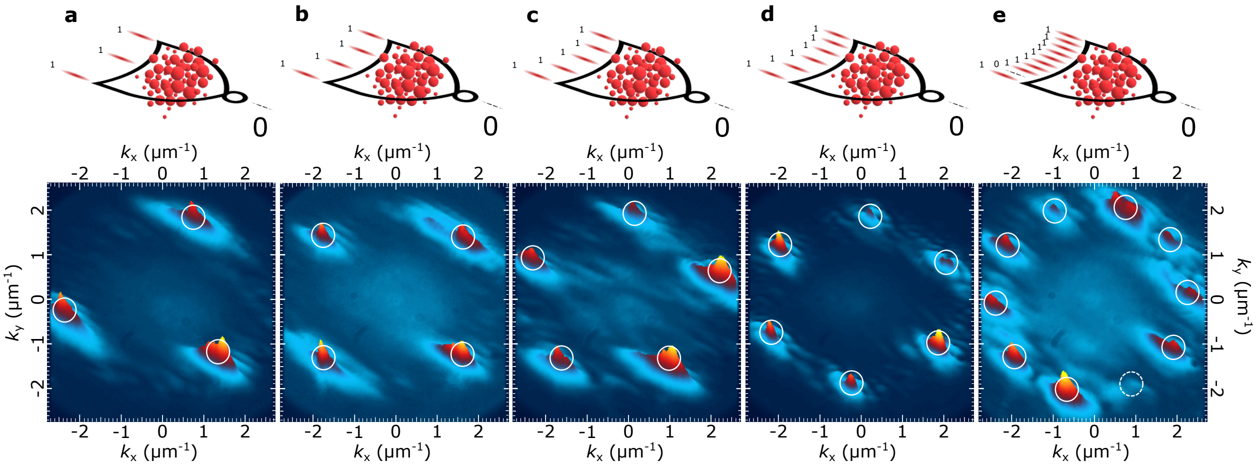

FIG. S7. **Multi-input NOR gate schematics and corresponding operation. a-d**, Three-, four-, five-, and six-inputs configurations in the presence of all control pulses. **e**, Ten-inputs NOR gate operation in the presence of nine out of ten control pulses.

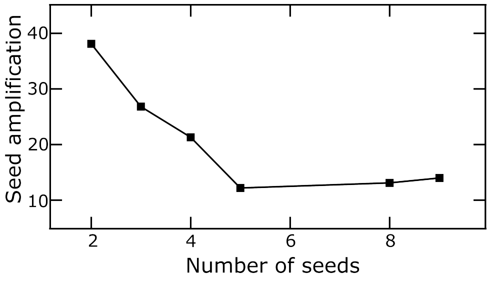

FIG. S8. **Seed amplification.** Dependence of the average seed amplification on the number of control-seed pulses.

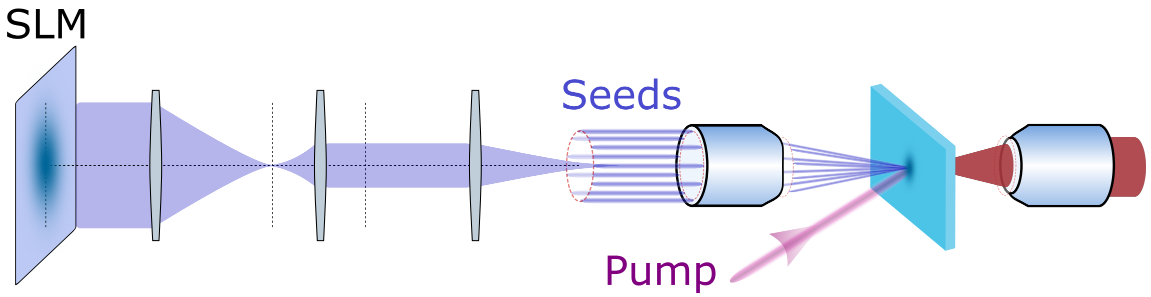

FIG. S9. **Experimental setup configuration for multi-input NOR gate.** Spatial Light Modulator (SLM) modifies the phase front of an incident collimated Gaussian beam profile according to prepared phase masks of concentrically equidistant Gaussian spots in real-space. Real-space image of the first order SLM's reflected phase plane is then conjugately projected on the objective aperture, which in turn focuses seed pulses on the sample in a single spot of ~ 25 μm in diameter resonantly injecting polaritons at equidistant in-plane wavevectors of the k-space ring of $|\mathbf{k}_{\parallel}| \approx 2.55$ μm^{-1}. Non-resonant pump pulse at 45° injects a hot exciton reservoir.

VI. NOR OPTICAL GATES

In this section, we provide a brief overview of the most advanced NOT/NOR optical gates found in the literature. Several platforms have been developed for optical circuitry to date. Generally, all of them rely on nonlinear optical phenomena modulating an optical output by means of weak optical control signal input. We deliberately do not include any interferometric configurations. As being phase-sensitive, they impose additional stringent requirements discussed in the main text. It is reasonable to divide all-optical universal gates in two large general classes: 1 - based on semiconductor optical amplifiers (SOA), 2 - other platforms, including fiber and wave-guide technologies.

Gates based on SOA. SOA-based optical gates usually rely on cross-gain (XGM) or cross-phase (XPM) modulation. For the former principle, one needs to realise the multi-mode regime when a particular mode amplification is decreasing with an increase of input signal intensity. This is possible once optical inputs are cross-coupled such that an effective altering of the gain in particular modes is achieved. Evidently, the NOR gate operation is typically achieved for co-propagating and counter-propagating beams [7, 8]. While the XGM deals with the imaginary part of nonlinear susceptibility, cross-phase modulation implies a change in the refractive index through the free carriers density modulation that modifies the dispersion of an amplifier active medium. Therefore, the other co-propagating beam undergoes spectral chirp, making negation functionality feasible at high repetition rates. Properly filtering the output signal, NOR gates have been realised allowing for high-speed signal processing as high as 40 Gb/s [9].

Alternative platforms. Optical fibers can be used to implement negation functionality and universal gate operation. According to the approach considered in [10], one can process the optical signals propagating in a fiber through specific clock pulses propagating along the same fiber but having a slightly different frequency. Via the process of non-degenerate four-wave mixing (NFWM) new components of the signal beam are formed, effectively reducing the initial intensity. Following this way, an all-optical NOT gate has been successfully implemented. Another nonlinear effect utilized for the negation functionality of an output optical signal in birefringent fibers is nonlinear polarization rotation (NPR). A linearly polarized pump beam induces a nonlinear phase shift between two polarization axes of a birefringent fiber. Along with the pump beam, an additional probe beam is injected, having the polar-

ization vector rotated by 45° with respect to the pump. When the intensity of the pump beam is adjusted properly, the probe polarization vector undergoes 90° rotation. Therefore, by placing a polarizer in the output port, one can observe NOR gate functionality, as was reported in [11]. One of the major drawbacks of these approaches is the necessity to cope with dispersive pulse broadening, which significantly limits the speed of signal processing. Moreover the high optical intensities required together with the latency and the footprint make fibers ineffective for such applications. Alternatively, silicon wire waveguides with strong optical confinement can be utilized. The small effective modal area (<0.1 μm^2) reduces the intensity for nonlinear control signals. In [12] authors harness two-photon absorption (TPA) in silicon to build an all-optical NOR gate. According to [12] two encoded signal pulses being injected in the waveguide allow for modulation of a continuous wave light. Relying on this principle, a NOR gate with 80 Gb/s operational speed has been demonstrated. One of the commonly-recognized limiting factors of the approach is an accumulation of free carriers due to the TPA process [12]. Injection-locking of a Fabry-Perot diode laser is another well-developed way to build all-optical negation logic. Normally, there is a dominant lasing mode. However, once signal beams are properly injected into the cavity, it may result in the collapse of the lasing from the dominant mode to another laser mode accordingly, thus reducing the major mode intensity. Such design allows for NOR/NOT gates operation with an extremely high extinction coefficient (over 40 dB). An apparent drawback of the approach relates to a long relaxation time of population inversion required for any conventional laser. It limits the speed of signal processing to a level of 10 Gb/s [13].

All the parameters of the NOT/NOR optical gates described above are summarized in Table S1. We note, that an extinction ratio for the CMOS logic gate is ≈ 3.7 dB [14].

TABLE S1. **The list of the reported NOR/NOT optical gates based on various platforms.**

Ref.	Nonlinear element	Modulation source	Response period (ps)*	Extinction ratio (dB)	Switching energy **
This work	Organic microcavity	Polariton condensation	∼0.5	≈18	∼10 pJ (80 fJ)***
[7]	SOA	XGM	>10	10	150 fJ
[9]	SOA	XGM/XPM	25	12	12 fJ
[10]	Optical fiber	FWM	∼ 12.5	10	-
[11]	Optical fiber	NPR	100	-	-
[12]	Si waveguide	TPA	12.5	10	∼8 pJ
[13]	Fabry-Perot diode laser	Injection-locking	25	40	30 fJ

* The response period is derived from the reported or theoretical prediction (where exists) operational speed

** The value corresponds to the input optical energy of a beam modulating a gate output

*** The value corresponds to the extinction ratio of 3 dB

VII. CASCADABILITY OF THE GATE

The signal regeneration mechanism is based on the polartion amplification [4] and the effect of the blueshift in organic semiconductors [6]. To realise this concept we direct the output of the first gate to a different spot on the sample at normal incidence so it is resonant with the ground-state mode of the lower polariton branch at this spot. In the presence of a non-resonant pumping pulse at the same position and at a necessary time-delay, we stimulate the ground-state polariton amplification that is controlled by both the power of the pumping pulse and the power of the seed (output of the previous gate). Since gate output logic-states of “1” and “0” rely on two different polariton condensate occupancies in the ground-state, the gate output power has two fixed values, and, therefore, the pumping parameters of the regeneration stage should be set to generate the output of the required amplitude and spectral tuning that matches the input of the subsequent gate at one input power value (“1”), and sufficiently different output at another input power value (“0”). We plot the normalized emission spectrum from the signal regeneration stage (for the case when the output of the first gate is in logic state “1”) in Fig.S10b (in red) together with the non-ground state normalized emission spectrum (in green). The regenerated signal then matches the desired parameters for the gate input providing a much better cascadability potential at the cost of one additional stage.

It is noteworthy that the present free-space optical setup allows for the proof-of-concept but is not scalable to a large number of gates. Therefore, to address the issue of scalability, one would need to explore a new architectures for integrated polariton transistors. There is ongoing work on lateral microcavities consisting of in-plane high contrast gratings that enable in-plane coupling of successive polariton gates [1].

[1] https://cordis.europa.eu/project/id/899141

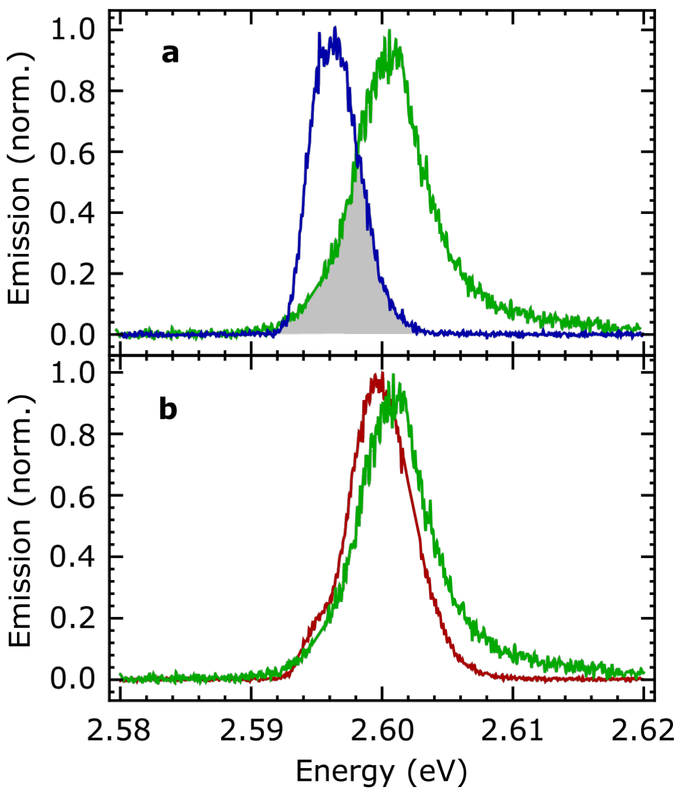

FIG. S10. **Cascadability of the universal gate.** **a**, The blue curve indicates the spectrum of emission from the ground state of the sample (the output of the gate). The green curve represents the emission from the non-ground state when the seed beam is present. The shaded area which is $\approx 50\%$ of the output signal enables effective seeding of the next subsequent gate. **b**, The comparison of the regenerated signal (red) with the non-ground-state emission profile (green).

[1] Anton V. Zasedatelev *et al.* Single-photon nonlinearity at room temperature. *Nature* **597**, 493–497 (2021).

[2] Shishkov,V. *et al.* Exact Analytical Solution for the Density Matrix of a Nonequilibrium Polariton Bose-Einstein Condensate. *Phys. Rev. Lett.* **128**, 065301 (2022).

[3] Plumhof, J. D., Stöferle, T., Mai, L., Scherf, U. & Mahrt, R. F. Room-temperature bose-einstein condensation of cavity exciton-polaritons in a polymer. *Nat. Mater.* **13**, 247-252 (2014).

[4] Zasedatelev, A.V. *et al.* A room-temperature organic polariton transistor. *Nat. Photon.* **13**, 378 (2019).

[5] Dai, D. & Monkman, A. Femtosecond hot-exciton emission in a ladder-type π-conjugated rigid-polymer nanowire. *Physical Review B* **87**, 045308 (2013).

[6] Yagafarov, T. *et al.* Mechanisms of blueshifts in organic polariton condensates. *Commun. Phys.* **3**, 18 (2020).

[7] Kumar, S. & Willner, A.E. Simultaneous four-wave mixing and cross-gain modulation for implementing an all-optical XNOR logic gate using a single SOA. *Opt. Express* **14**, 5092–5097 (2006).

[8] Son, C.W. *et al.* Realisation of all-optical multi-functional logic gates using semiconductor optical amplifiers. *Electron. Lett.* **42**, 1057–1058 (2006).

[9] Dong, J., Zhang, X., Xu, J. & Huang, D. 40 Gb/s all-optical logic NOR and OR gates using a semiconductor optical amplifier: Experimental demonstration and theoretical analysis. *Opt.Commun.* **281**, 1710–1715 (2008).

[10] Lai, D.M.F., Kwok, C.H. & Wong, K.K.-Y. All-optical picoseconds logic gates based on a fiber optical parametric amplifier. *Opt. Express* **16**, 18362–18370 (2008).

[11] Wang, W. *et al.* Experimental research on 10 Gb/s all-optical logic gates with return-to-zero data in high nonlinear fiber. *Asia Communications and Photonics Conference and Exhibition,* 830829 (2011).

[12] Liang, T.K. *et al.* High speed logic gate using two-photon absorption in silicon waveguides. *Opt. Commun.* **265**, 171–174 (2006).

[13] Uddin, M.R., Lim, J.S., Jeong, Y.D. & Won, Y.H. All-optical digital logic gates using single-

mode Fabry–Perot laser diode. *IEEE Photon. Technol. Lett.* **21**, 1468–1470 (2009).

[14] Uyemura, J.P. CMOS Logic Circuit Design *Springer US* (1999).
